# Influence of high-resolution data on the assessment of forest fragmentation

**DOI:** 10.1007/s10980-019-00820-z

**Published:** 2019-09-01

**Authors:** J. Wickham, K. H. Riitters

**Affiliations:** National Exposure Research Laboratory, Office of Research Development, U.S. Environmental Protection, Agency, 109 T.W. Alexander Dr.; MD: 343-05, Research Triangle Park, NC 27711, USA; Southern Research Station, United States Department of Agriculture, Forest Service, Research Triangle Park, NC 27709, USA

**Keywords:** Chesapeake Bay land cover, Forest spatial patterns, NLCD, Spatial resolution remote sensing

## Abstract

**Context:**

Remote sensing has been a foundation of landscape ecology. The spatial resolution (pixel size) of remotely sensed land cover products has improved since the introduction of landscape ecology in the United States. Because patterns depend on spatial resolution, emerging improvements in the spatial resolution of land cover may lead to new insights about the scaling of landscape patterns.

**Objective:**

We compared forest fragmentation measures derived from very high resolution (1 m^2^) data with the same measures derived from the commonly used (30 m × −30 m; 900 m^2^) Landsat-based data.

**Methods:**

We applied area-density scaling to binary (forest; non-forest) maps for both sources to derive source-specific estimates of dominant (density ≥ 60%), interior (≥ 90%), and intact (100%) forest.

**Results:**

Switching from low- to high-resolution data produced statistical and geographic shifts in forest spatial patterns. Forest and non-forest features that were “invisible” at low resolution but identifiable at high resolution resulted in higher estimates of dominant and interior forest but lower estimates of intact forest from the high-resolution source. Overall, the high-resolution data detected more forest that was more contagiously distributed even at larger spatial scales.

**Conclusion:**

We anticipate that improvements in the spatial resolution of remotely sensed land cover products will advance landscape ecology through reinterpretations of patterns and scaling, by fostering new landscape pattern measurements, and by testing new spatial pattern-ecological process hypotheses.

## Introduction

Landscape ecology was born out a new technology—remote sensing. Carl Troll (1899–1975) is credited with the insight because of his observation that the image in an aerial photograph was a picture of an ecosystem (Troll 1971). As he points out in his 1971 paper, Troll introduced the concept of landscape ecology in 1939 (Troll 1939; see also Butzer 2004). Aerial photography was emerging as a new technology in 1939, recognized for its military applications and, somewhat later, for its academic value (Estes et al. 1980).

Landscape ecology found its way to the United States by the 1980s (Risser et al. 1984), and similarly relied on remotely sensed data to articulate its main concepts. *Indices of landscape pattern* (O’Neill et al. 1988), still the most-cited paper in the journal *Landscape Ecology* (Wu 2013), used digital land cover maps derived from interpretation of aerial photography to show how measures commonly applied in ecological field studies (e.g. Brower and Zar 1977) could be used to provide ecological insight over a much broader geographic area using a domain of ecological organization other than species. The paper stimulated an enthusiastic investigation of landscape pattern, including a wide array of pattern measurements as well as software to calculate the measurements (Turner et al. 1989; Baker and Cai 1992; McGarigal and Marks 1995; Riitters et al. 1995)

Remote sensing advanced as landscape ecology developed. The paper by O’Neill et al. (1988) used land cover from the U.S. Geological Survey (USGS) Land Use Data Analysis (LUDA) program. LUDA was based on high-altitude aerial photography that had a 4-ha minimum mapping unit (mmu) for urban classes and a 16-ha mmu for all other classes (Loveland 2012). The data were eventually converted to raster format (Loveland 2012) at a 4-ha pixel^−1^ spatial resolution (Fegeas et al. 1983). The raster formatted LUDA data were the basis of the research reported by O’Neill et al. (1988) and others (e.g., Riitters et al. 1995; Turner et al. 1989). By the late 1990s, through the formation of the multi resolution land characteristics (MRLC) consortium (Homer et al. 2004; Wickham et al. 2014; Yang et al. 2018), remote sensing of land cover had advanced to producing land cover for the conterminous United States from the Landsat satellite series (Vogelmann et al. 2001; Homer et al. 2007), which had a native resolution of 0.09 ha pixel^−1^. The 4500% increase in spatial resolution increased confidence in the assumption of homogeneity for a pixel’s land cover class label and obviated the need for “mixed” classes (e.g., cropland and natural vegetation) that were typically necessary at coarser spatial resolutions (e.g., Loveland et al. 2000). The wider availability of higher resolution land cover data permitted more meaningful measurements of landscape patterns for riparian zones, urban areas, and feature (e.g., forest) connectivity (Jones et al. 1997; Wickham et al. 1999). Examples of new insights attributable to the availability of higher resolution land cover data distinguished forest edge, interior, and perforations for the conterminous United States (Heilman et al. 2002; Riitters et al. 2002). The higher resolution permitted more accurate overlays with ancillary data such as road maps (Heilman et al. 2002) and better characterization of the components of fragmentation (Riitters et al. 2002). The pattern metrics derived from the land cover data were subsequently included in national ecological assessments such as the Montréal Process (Riitters et al. 2004) and Heinz Center (2008).

Nearly two decades have passed since measurement of landscape indicators from Landsat-based land cover maps became commonplace, and we may be on the cusp of another significant technological advance. The United States Department of Agriculture (USDA), Farm Service Agency (FSA) now provides raster images at 1 m^2^ spatial resolution for the United States through its National Agriculture Imagery Program (www.fsa.usda.gov/programs-and-services/aerial-photography/imagery-programs/naip-imagery/). Use of NAIP imagery for land cover mapping and other applications is widespread (Popkin 2018). In addition, the National Oceanic and Atmospheric Administration (NOAA), Coastal Change Analysis Program (C-CAP) plans to map land cover from NAIP for the coastal portions of the United States (N. Herold pers. comm.; coast.noaa.gov/digitalcoast/data/), and NAIP-based land cover is available for about 25 U. S. metropolitan areas through the Environmental Protection Agency (EPA), EnviroAtlas project (www.epa.gov/enviroatlas). Because of ever-increasing computing capability (both desktop and cloud) and continued advances in remote sensing technology, it is likely that land cover from high resolution sources will become the preferred choice in the future, replacing land cover data from Landsat and similar satellites (e.g., Sentinel-2).

The objective of this paper is to provide a glimpse into that future as it relates to measurement of landscape pattern. We compare measurements of forest fragmentation (Riitters et al. 2002) derived from high-(1 m^2^ pixel^−1^) and low-resolution (900 m^2^ pixel^−1^) data (hereafter, fine grain and coarse grain, respectively). NLCD 2011 land cover data (Homer et al. 2015) were used as the coarse-grain dataset and land cover data derived from NAIP for the Chesapeake Bay region (chesapeakeconservancy.org/conservation-innovation-center/high-resolution-data/land-cover-data-project/) were used as the fine-grain (i.e., 1 m^2^ pixel^−1^) dataset. The behavior of landscape pattern measures across a range of grain sizes has been the topic of several studies (e.g., Turner et al. 1989; Corry and Lafortezza 2007; Buyantuyev et al. 2010; Wu 2013). Here we specifically test the assertion in Riitters et al. (2002) that forest fragmentation would be more severe if the analysis had been undertaken with finer grain land cover data. The rationale was that the greater detail available from a finer grain will improve detection of interruptions in the forest canopy, thereby reducing forest density where forest is abundant, and, by extension, increase forest density where forest is less abundant. The shift in forest patterns expected from improved spatial resolution has broad implications for landscape assessments and ecological interpretation of landscape data. Notwithstanding map accuracy issues, more perforated interior forest, realized from an assessment based on finer grain data (Foreman 1995), may trigger changes in forest management locally or regionally (Riitters et al. 2018). Similarly, increases in the amount of forest in exurban to urban contexts may have implications for water quality management (Claggett et al. 2013) and spatial variation in the magnitude of the urban heat island (UHI) effect (Quattrochi and Ridd 1996).

The concept of “forest” is both intuitive and ambiguous. Comparison of fragmentation from 1 m^2^ - pixel^−1^ (0.0001 ha pixel^−1^) and 900 m^2^ pixel^−1^ (0.09 ha pixel^−1^) land cover data may be questioned because the grain size of the former represents individual trees rather than forests. However, definitions of what constitutes a “forest” depend on the objective of the assessment or analysis (Chazdon et al. 2016). Not all definitions of forest include a minimum area, and those that do are not consistent in their minimum area threshold. Our view is that the comparison is valid because forest minimum area criteria vary with the ecological question (Kapos 1989; Wiens and Milne 1989). Furthermore, the minimum area criterion for the UN Framework Convention on Climate Change (UNFCCC) is 0.05 ha (Chazdon et al. 2016), and the remote sensing rule-of-thumb is that the spatial resolution of the sensor must be smaller than a feature for it to be detected accurately (https://www.nrcan.gc.ca/node/9407). Thus, a sensor with a 0.0001 ha pixel^−1^ spatial resolution would be more appropriate than a sensor with a 0.09 ha pixel^−1^ to map forests as small as 0.05 ha.

## Methods

**Fig. 1:**
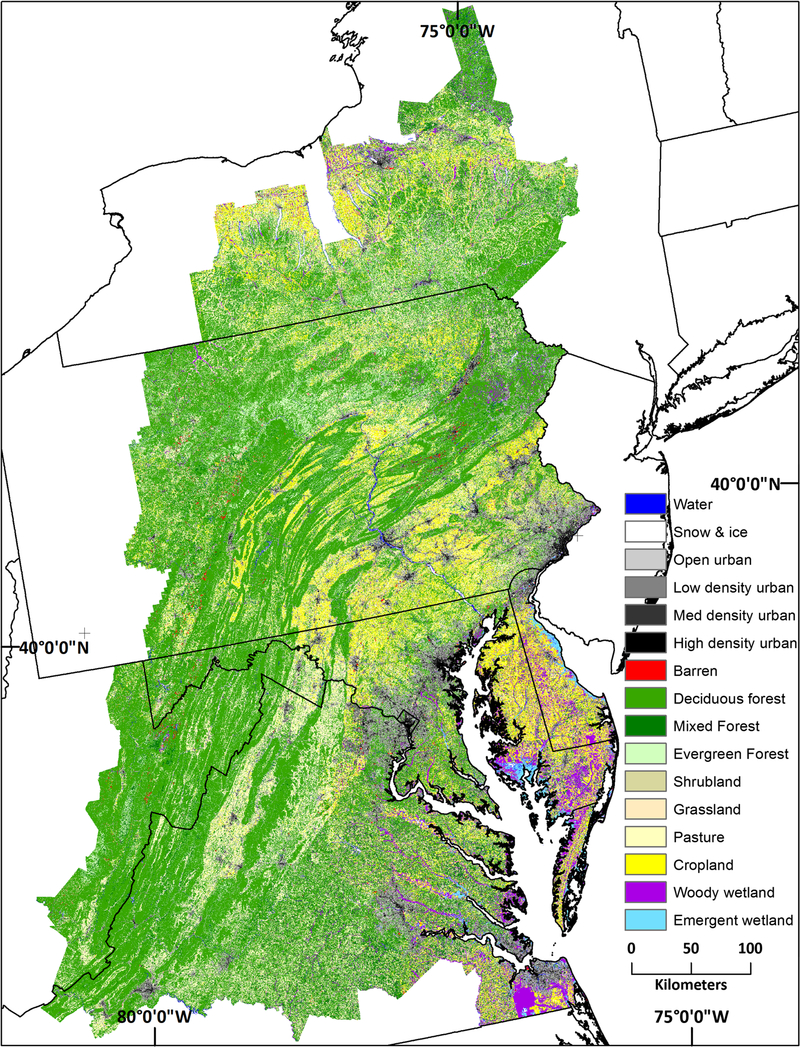
NLCD 2011 land cover for the Chesapeake Bay region

Comparison of forest fragmentation measurements from fine- and coarse-grain land cover datasets was undertaken in the 248,000 km^2^ Chesapeake Bay region (Fig. 1) because of the availability of NAIP-based (1 m^2^) land cover (see URL in the “Introduction”). The fine-grain land cover mapping effort was sponsored by the Chesapeake Bay Program (www.chesapeakebay.net) to support improved water-quality modeling of the Chesapeake Bay. The six-class dataset, produced from 2013 NAIP imagery and ancillary data, included water, barren, tree canopy and shrubs, herbaceous, impervious (roads), and impervious (other). User’s and producer’s accuracies for the tree canopy and shrubs class were 83% and 81%, respectively (Pallai and Wesson 2017). The NLCD 2011 land cover data, nominally 2 years older than the Chesapeake Bay land cover data, were clipped and aligned to the Chesapeake Bay land cover dataset for the comparison. The pixels in both land cover datasets were then reclassified into forest and non-forest (forest = 1; non-forest = 0). The forest class for the Chesapeake Bay land cover data was the tree canopy and shrub class; all other classes were masked (set to null). Deciduous forest, evergreen forest, mixed forest, and woody wetlands comprised the forest class the NLCD 2011 land cover. User’s and producer’s accuracies for the NLCD 2011 three upland forest classes were 94% and 88%, respectively, and the corresponding values for the woody wetlands class were 74% and 87%, respectively (Wickham et al. 2017). Total forest area was approximately 16.4 × 10^6^ ha based on the fine-grain (Chesapeake Bay) land cover data and 14.8 × 10^6^ ha for the coarse-grain (NLCD) land cover data.

We used forest density (Fd) as our measure of forest fragmentation. Forest density was estimated for five window sizes using the binary (forest or non-forest) land cover maps by summing the number of pixels labeled as forest in a given window and assigning the result to the center pixel in the window. The square window side lengths in meters (m) were 150, 270, 810, 2430, and 7290, which is equivalent to side lengths of 5, 9, 27, 81, and 243 in pixels for the NLCD 2011 land cover data. The areas of the window sizes were 2.25 ha, 7.29 ha, 65.61 ha, 590.49 ha, and 5314.41 ha, respectively. An additional 1 m of side length was added to each of the window sizes for the Chesapeake Bay land cover data so that windows had a clearly defined center pixel. Results are reported only for pixels labeled as forest in the original land cover maps, i.e., forest density for forested locations rather than forest density for all locations (Riitters et al. 2002).

For each forest location and at each spatial scale (window size), we report the area of forested locations meeting or exceeding eight forest density thresholds from 40 to 100% in 10% increments and 95%. The range of thresholds provide a convenient and flexible means for interpreting forest connectivity and fragmentation. We used the thresholds ≥ 60%, ≥ 90%, and 100% to define “dominant,” “interior,” and “intact” forest classes, respectively. There were different levels of precision available for defining the thresholds because of the different spatial resolutions of each land cover dataset. The number of pixels for each forest density threshold at each scale was determined by multiplying the number of pixels in the window by the specified percentage and then rounding up to the next integer value when the result was a real number. Rounding up, regardless of whether the fractional portion of the threshold was greater than or equal to 0.5, ensured that the density threshold was met.

## Results

There was substantially less intact forest (100% threshold) identified by the fine-grain land cover data across all spatial scales examined (Table 1). The substantial reduction of intact forest resulting from the change in grain size occurred even though the increase in spatial resolution resulted in an increase in total forest area (see “Methods”). At the smallest spatial scale (2.25 ha), the area of intact forest from the coarse-grain data exceeded the area of intact forest from the fine-grain data by more than 1.3 million hectares (Table 1). The magnitude of the differences of coarse-minus fine-grain estimates declined to approximately 36,600 ha at the 590.49-ha scale, as the requirement of uninterrupted forest became harder to attain with increasing spatial scale for both coarse- and fine-grain data. Intact forest did not occur in either land cover dataset at the 5314.41-ha scale.

**Fig. 2:**
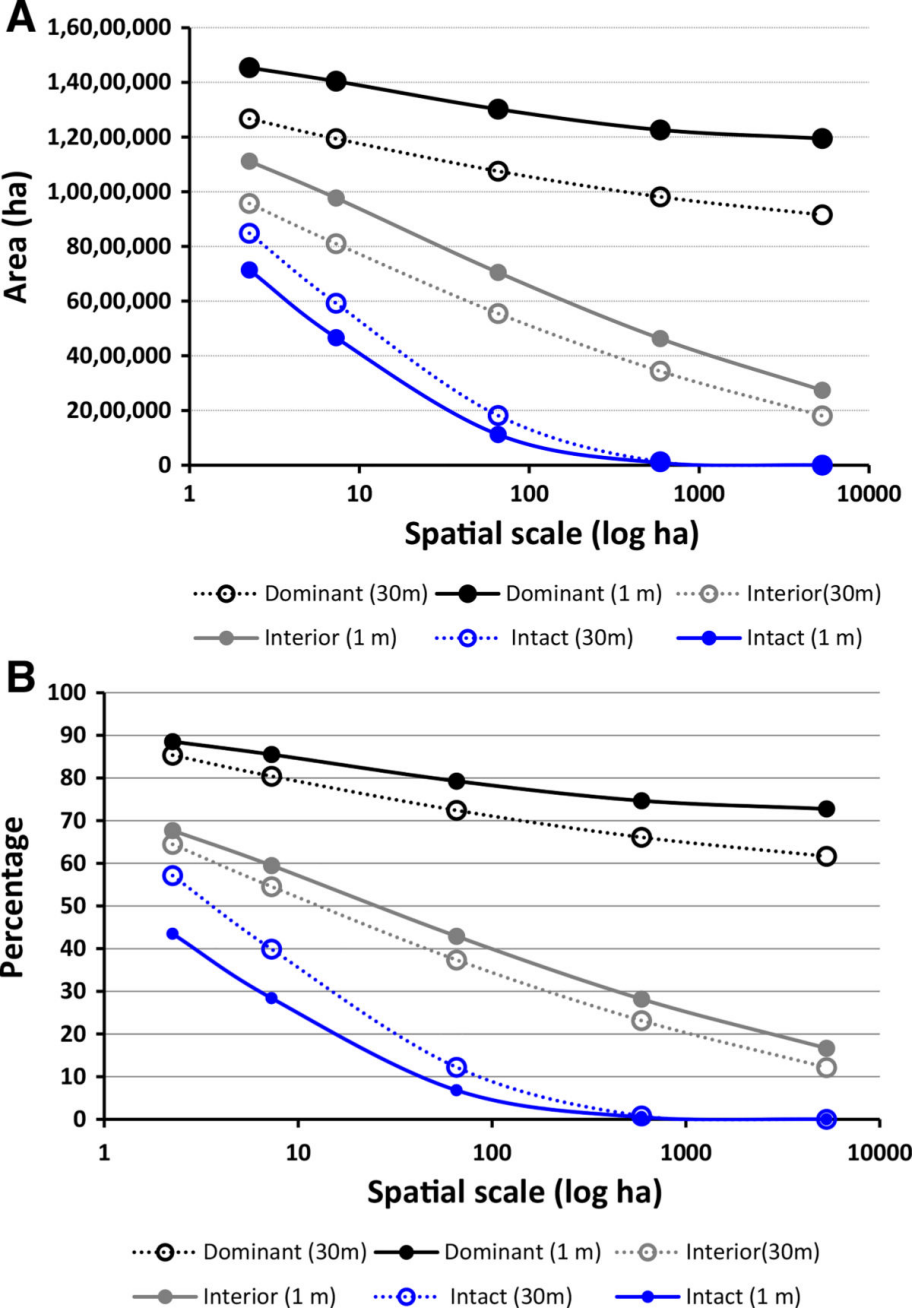
Forest density class **a** area and **b** percentage at five spatial scales and two grain sizes

The substantial reduction in intact forest that resulted from switching from coarse- to fine-grain land cover data was accompanied by a substantial increase in the percentage and amount of interior forest (≥ 90% threshold). The amount of interior forest detected using the fine-grain land cover data was greater than the amount detected by coarse-grain land cover data by more the 900,000 ha across all window sizes (Table 1). This pattern remained for a more conservative interior forest threshold (≥ 95%). Overall, the fine-grain estimate indicated that there was more forest that was more contagiously distributed even at larger spatial scales (Fig. 2). Excluding intact forest, the fine-grain estimates of percentage of dominant and interior forest exceeded their coarse-grain counterparts across all spatial scales.

**Fig. 3:**
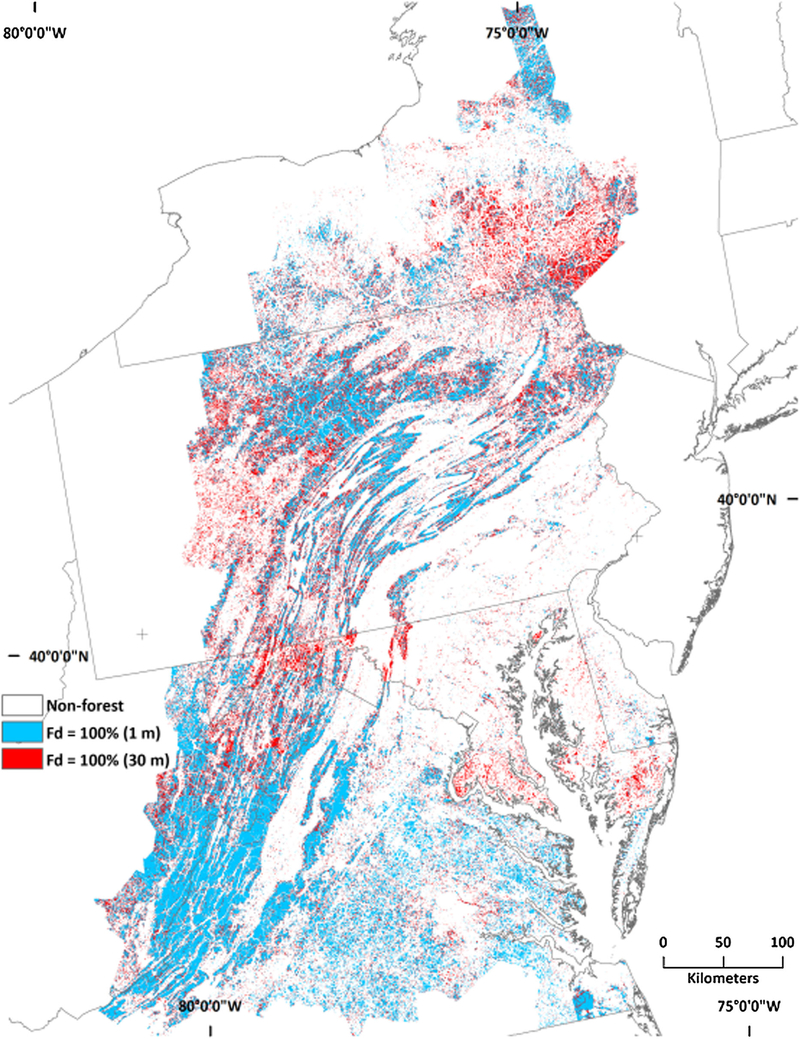
Spatial distribution of intact forest (forest density (Fd) = 100%) at the 7.29-ha scale (“+” = 40°N, 75°W; 40°N 80°W)

**Fig. 4:**
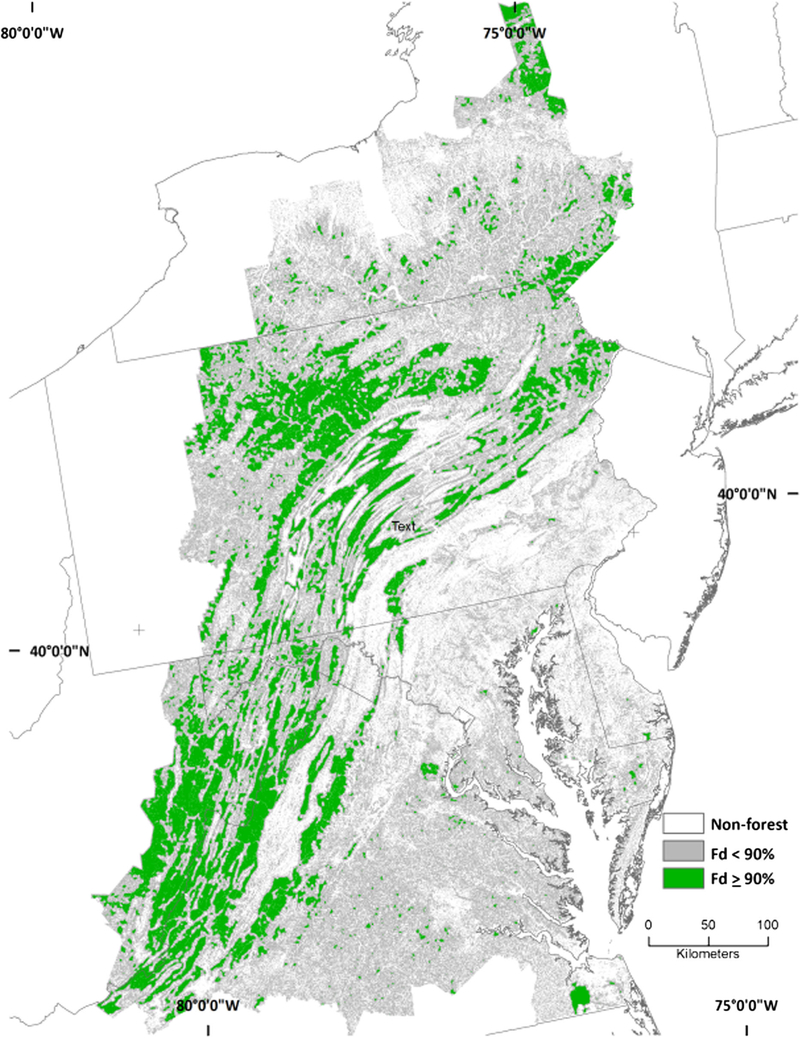
Interior forest (Fd ≥ 90%) from NLCD at 590.49-ha scale

**Fig. 5:**
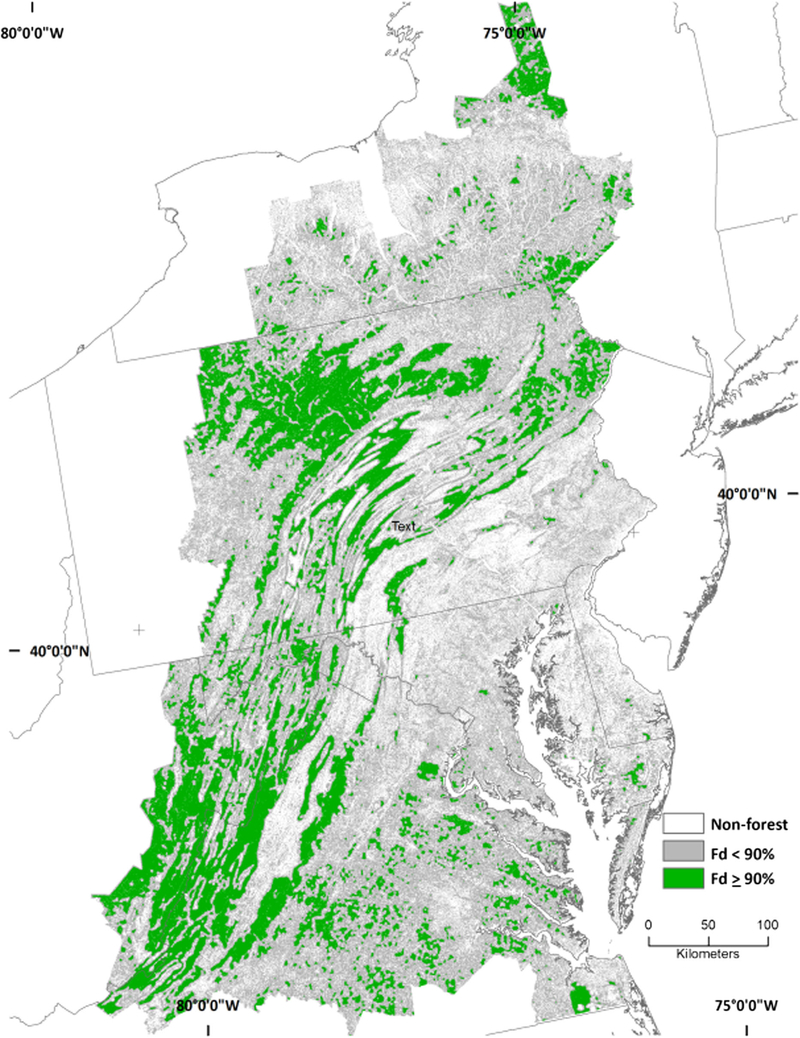
Interior forest (Fd ≥ 90%) from 1 m^2^ land cover data at 590.49-ha scale

**Fig. 6:**
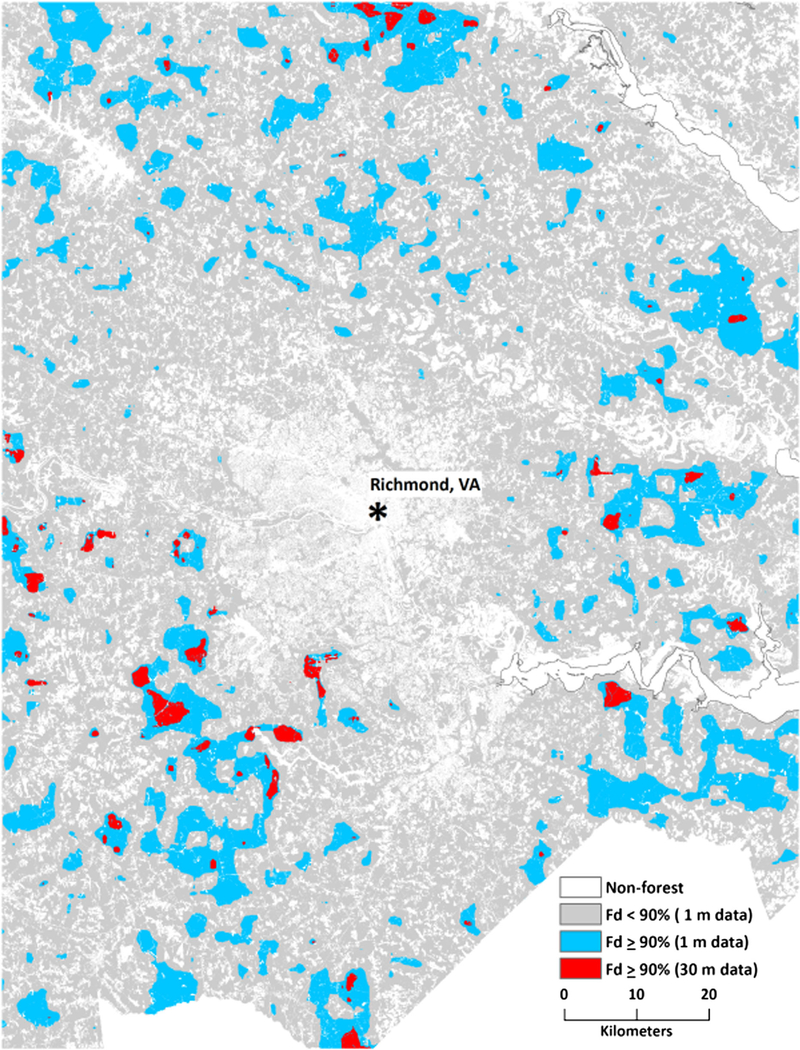
Fine- and coarse-grain interior forest (Fd ≥ 90%) in the vicinity of Richmond, VA

The area differences between fine- and coarse-grain forest spatial patterns were accompanied by geographic differences. Intact forest was less predominant in the Appalachians and elsewhere in the fine-grain data because the fine-grain data detected non-forest features “invisible” to the coarse-grain data (Fig. 3). The ability of the fine-grain land cover data to detect features “invisible” to the coarse-grain data also resulted in detection of forest missed at the coarser spatial resolution, which contributed to the substantial increase in the amount of interior forest (along with a decline in intact forest) across all spatial scales (Table 1; compare Figs. 4, 5). There was a considerable amount of interior forest outside that the Appalachian region that was not detected by the coarse-grain data at the larger spatial scales (Fig. 6; compare Figs. 4, 5).

## Discussion

Despite uncertainties in the future of the USDA NAIP program (Popkin 2018), it is plausible that land cover from high resolution sources will be commonplace across the United States in the near future, supplanting land cover data from Landsat and other platforms with similar spatial resolution. It is likely that such an increase in spatial resolution will influence what we see, how we measure what we see, and how we interpret what we measure (Mandelbrot 1982). In this study, using a previously established measurement method (forest density from spatial convolution), we found that use of finer grain maps influenced both what we saw and our interpretations of what we saw. By switching from coarse- to fine-grain resolution we found that forest fragmentation was less severe rather than more severe, except for the intact forest class, and that interior forest was more uniformly distributed across the region. The earlier improvement in resolution from 4 to 0.09 ha pixel^−1^ permitted meaningful quantification of landscape features (Jones et al. 1997; Riitters et al. 2002; Vogt et al. 2007; Wickham et al. 2010) that were recognized earlier (Forman and Gadron 1986; Zipperer 1993) but less meaningful when measured using land cover data with 4-ha pixel^−1^ spatial grain. Apart from improved spatial precision of pattern indices, another substantial increase in spatial resolution (e.g., 0.09 ha pixel^−1^ to 0.0001 ha pixel^−1^) may result in the identification and measurement of new landscape patterns and indices. The amount of bare ground, an important indicator of rangeland condition in arid and semi-arid environments (Booth and Tueller 2003; Breckenridge and Dakins 2013), is one example of a well-established metric whose quantification would become more feasible and meaningful with higher resolution land cover data.

New applications of landscape pattern analysis are another aspect of a future landscape ecology fostered by finer grain land cover data. Urban ecology is one example where finer grain land cover data are already being used to link spatial pattern and process. Several studies that have used high-resolution land cover data have found an inverse correlation between urban vegetation and the magnitude of the Urban Heat Island (UHI) effect and human heat stress (Zhou et al. 2011; Jenerette et al. 2016; Li et al. 2016). Use of high-resolution land cover in urban settings extends beyond linkages between spatial patterns of vegetation and surface temperatures, including linkages between spatial patterns of urban vegetation and spatial patterns of socio-economic factors (e.g., income, ethnicity) (Schwarz et al. 2015); mapping of grasslands around airports to identify areas suitable for alternative energy production (DeVault et al. 2012); the effect of shifting proportions of trees and grass on urban watershed management (Claggett et al. 2013; Beck et al. 2016); spatial association between urban vegetation and disease vectors (Landau and van Leeuwen 2012); and the identification of new landscape features characteristic of urban settings that are difficult to detect at the spatial resolution of Landsat (Singh et al. 2018). Some other examples of spatial pattern detection that can be undertaken with improved spatial resolution include shrub encroachment (Davies et al. 2010), bare ground identification in arid and semi-arid environments (Breckenridge and Dakins 2013), and impacts of shale gas extraction on forest spatial patterns (Drohan et al. 2012).

The interaction between spatial pattern and ecological process has been a motivating concept and a defining principle of landscape ecology (Gustafson 2018; Turner 1989). Perhaps less well recognized is the interaction between pattern and policy. What surrounds a park or refuge (landscape context) is as germane to their management as their contents (Gross et al. 2009; Jones et al. 2009). Others have also shown management of the landscape may be a more effective means of promoting forest sustainability than management of the forests themselves (Riitters et al. 2009). The differences between fine- and coarse-grain estimates of dominant, interior, and intact forest reported here are perhaps most relevant to how they might contribute to forest management and policy issues. It is possible that the more conservative, fine-grain estimate of remote, roadless, intact forest (Riitters and Wickham 2003) will motivate a renewed look at preservation in the Appalachians and elsewhere. It is also possible that the improved estimates of interior forest outside that Appalachian region could motivate interest in management of forest from a landscape perspective (Thompson 2006; Riitters et al. 2009).

We searched all issues *Landscape Ecology* for the term NAIP, and it appeared in 19 papers. Five of the 19 papers, all published since 2016, used NAIP data as a source of land cover to examine relationships between spatial pattern and ecological processes (Berg et al. 2017; Jenerette et al. 2016; Miller et al. 2017; Shahan et al. 2017; White et al. 2018). Here, we used high-resolution land cover to confirm a hypothesis about the effect of landscape grain on forest fragmentation patterns and briefly discussed the implications (management and policy relevance) of the results. We expect that future landscape analyses will rely routinely on land cover data derived from high resolution sources. The shift from Landsat-based land cover to high-resolution land cover will likely open the door to a wider range of spatial pattern-ecological process evaluations. If funding of NAIP continues, we would expect it to foster further advances landscape ecology.

## Figures and Tables

**Table 1:** (A) Fine- minus coarse-grain forest area difference (ha) and (B) coarse-grain forest area

Forest density (%)	Spatial scale				
	2.25 ha	7.29 ha	65.61 ha	590.49 ha	5314.41 ha
(A)					
≥ 40	2,083,356	2,285,500	2,522,520	2,682,915	3,255,486
≥ 50	2,064,895	2,202,949	2,409,862	2,554,352	3,185,795
≥ 60	1,865,597	2,100,951	2,267,121	2,447,152	2,794,899
≥ 70	1,920,957	1,963,893	2,096,673	2,267,732	2,583,227
≥ 80	1,600,523	1,801,353	1,852,927	1,830,003	1,724,465
≥ 90	1,547,598	1,670,675	1,500,392	1,193,555	933,480
≥ 95	896,156	1,252,675	1,289,377	807,657	499,474
100	− 1,341,996	− 1,259,004	− 694,544	− 36,622	0
(B)					
≥ 40	13,515,224	13,121,687	12,539,116	12,198,415	11,960,452
≥ 50	13,108,508	12,640,428	11,790,328	11,245,707	10,920,015
≥ 60	12,669,873	11,938,866	10,750,083	9,813,411	9,151,595
≥ 70	11,775,833	11,022,699	9,390,184	7,953,038	6,796,658
≥ 80	11,020,195	9,836,200	7,710,290	5,837,902	4,386,764
≥ 90	9,568,374	8,097,989	5,545,623	3,434,862	1,803,283
≥ 95	9,068,566	7,100,068	3,998,056	1,996,101	629,882
100	8,481,661	5,920,466	1,811,752	121,844	0
